# Secret Lanthanides

**Published:** 2014-09-25

**Authors:** CM Sturza

**Affiliations:** ArsMedica, Bucharest, Romania

**Keywords:** lanthanides, periodic table of the elements, element theory, homoeopathy, autoimmune diseases

## Abstract

Abstract

Lanthanides are a group of 15 chemical elements which, together with their salts, have come to be used in the last decade as homoeopathic remedies. The effective introduction of lanthanides and their salts into the clinical use, as homoeopathic remedies was based on the idea of Jan Scholten, MD to relate their physicochemical properties shown in the periodic table of elements to their homoeopathic potential. The lanthanides and their salts were prepared as homoeopathic remedies by Pharmacist Robert Münz.

## Introduction

The fact that lanthanides are the largest group in the periodic table was mentioned in a previous article [**1**]. They are highly electropositive and reactive metals. The spectral and magnetic properties of lanthanide ions are remarkable. Lanthanide complexes as well as their simple salts are used more and more in fields such as Biology, Biochemistry and Medicine.

 The purpose of this article is to present to medical and pharmaceutical specialists an event pertaining to the area of complementary medicine, specifically to homeopathic pharmacotherapy, that is, the introduction into clinical use, of an entire group of homeopathic remedies, the lanthanides and their compounds nearly ten years ago. We owe this event to a homeopathic doctor with chemistry background, Jan Scholten MD, and to a homeopathic pharmacist, Magister Robert Münz.

 The introduction into clinical use of lanthanides and their salts was based on the inspired, or should we say, genius idea of deriving their homeopathic potential from the chemical properties of lanthanides as they appeared in the classical periodic system, more precisely from the understanding of this system on the light of element theory (Scholten). 

## Discussion

 In order to understand the origins and the historical evolution of this phenomenon, we will draw a parallel between the Mendeleev’s classical periodic system of elements and the periodic table of the same elements in Jan Scholten’s vision [**3**].

 A periodic system of elements is an ordering of the chemical elements with the purpose of gaining knowledge about the substances that nature builds upon. This ordering, of course, requires a criterion.

 In 1869, the Russian chemist Dmitri Ivanovich Mendeleev (1834-1907) published the Principles of Chemistry, translated into English in 1891. This work formulated the law of periodicity of the properties of chemical elements. Based on this, Mendeleev established a classification of the elements, better known as the periodic system of elements. This scientific breakthrough was made possible by the previous discovery, in the late eighteenth and nineteenth century, of a large number of chemicals including: hydrogen (1766 Cavendish), oxygen (1771 Scheele), nitrogen (1772 Rutherford), chlorine (1774 Scheele), potassium, sodium (1807 Davy), calcium (1808 Davy), aluminum (1825 Ørsted).

 Mendeleev came up with the idea of arranging the elements in an order that presupposes increasing atomic mass. He noticed that, as the atomic masses increased, properties seemed to repeat at every eight elements. Based on this arrangement and the knowledge at that time, Mendeleev predicted the existence of other elements, which had yet to be discovered, and left free blanks in the periodic table. As foreseen, these gaps would later be filled by newly discovered elements. However, despite the firm conviction, at that time, that atomic mass should be the criterion in establishing the order of elements, Mendeleev’s good common sense and the obvious chemical properties of certain elements led him to accept three inversions within his own framework: Cobalt/Nickel, Tellurium/Iodine, Argon/ Potassium. This would eventually prove that the chemical properties of elements are determined by their electronic structure, only partially known at the time, and not by their atomic mass.

**Fig. 1:**
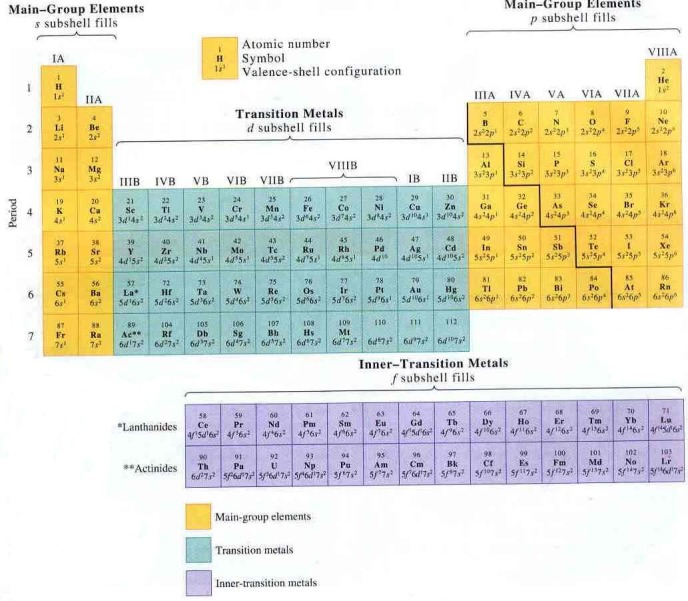
The periodic table of elements with groups and periods notation

**Fig. 2:**
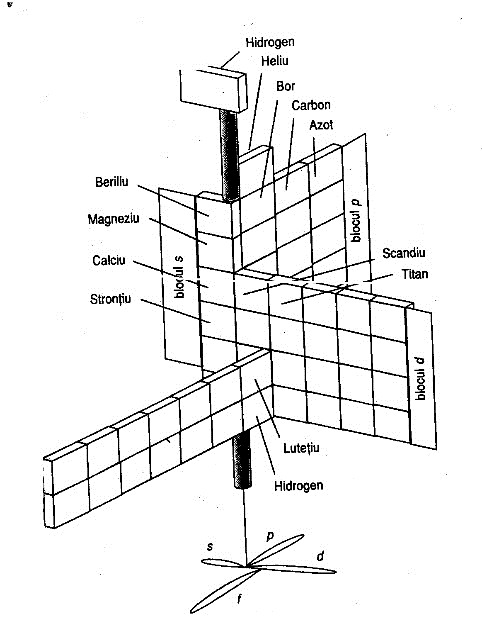
Paul Giguere 3D periodic table

**Fig. 3:**
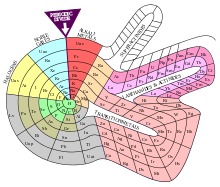
2D periodic table of elements

 The design of Mendeleev’s periodic table was reinterpreted over time and continues to be: different authors are proposing new layouts, according to various criteria that help us understand different aspects of the same reality. The figure below (**Fig. 1**) is a familiar layout found in all chemistry textbooks on the planet:

 Another version of the periodic table (**Fig. 2**) shows a three-dimensional view, based on filling the orbitals s, p, d, f. This image corresponds to the inner electronic structure of atoms. A flat image using curves is shown in Fig. 3.

**Fig. 4:**
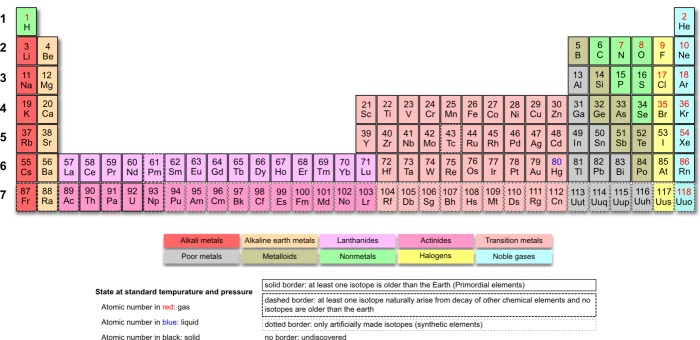
Niels Bohr’s version of the periodic table

 Niels Bohr's model, Fig. 4, respects the strict electronic configuration of elements and emphasizes the position of Lanthanum plus the 14 lanthanides between the main group II and subgroup III. 

 Both the periodic system and pharmacopoeia (as the acme of allopathic drugs and homeopathic remedies) are cultural products: the primary purpose of the former is knowledge, while the latter has a more practical purpose, namely a therapeutic one. No matter how far apart the two might seem at a first glance, their history, i.e. their transformation over time, brings out an interesting connection.

 Perhaps the most obvious, most magnificent, most spectacular and most practical proof of this connection is the relatively recent introduction into medical use, in a relatively short period of time, of a whole group of chemical elements, the lanthanides and their compounds. This is truly an unprecedented phenomenon in the history of homeopathic medicine.

 In allopathic medicine, owing to their spectral and magnetic properties, some of the lanthanides and their complexes make possible advances in today’s imaging, therefore having a purely diagnostic function [**1,2**].

 Lanthanides and some of their compounds have recently entered into regular use in homeopathic medicine and many homeopathic practices.

 Let us remember that homeopathy is built on the principle of analogy. This is why homoeopathy has enjoyed ever since its founding, an admirable consistency between clinical medicine and pharmacy. All materials used were tested and continue to be tested on healthy humans and thus knowledge of a material becomes congruent with its therapeutic use. As a result, the homeopathic diagnosis becomes one with the homoeopathic remedy.

 At this point, we consider it appropriate to firmly express our opinion, in a correct and well-documented manner, that is, the Pharmacon in homoeopathy should continue to be called remedy. Firstly, out of respect for the profoundly different nature of each of the two therapeutic approaches in question (allopathy and homoeopathy) and secondly, out of no less respect for the manner of preparation peculiar to each of them.

 Since its founding by Samuel Hahnemann, homoeopathy has benefited from a Materia Medica of its own. Over time, Materia Medica has come to be perceived by the collective mind of homoeopathy professionals as the registration of clinical aspects, while the homoeopathic Pharmacopoeia is seen as a collection of technical directions for preparation, otherwise scarce in number and invariable, and a precise description of the materials used and of the strains, which is the name of the materials to be produced.

 With all this in mind, let us return to our unprecedented phenomenon mentioned above and explain the way it occurs.

 It occurs through a deductive process applied by the mind of a homoeopath with chemistry background: Jan Scholten, MD. Thus, Dr. Scholten anticipated the Lanthanides’ therapeutic potential, in other words their clinical picture, based on their chemical characteristics as derived from their position in the Periodic Table of the Elements. The distinctive feature of the Lanthanides’ electronic structure is the gradual filling up with electrons (from 1 to 14) of a sublevel (subshell) that is well shielded from external interactions. This is the so-called 4f subshell that is progressively filled from cerium to lutetium in the presence of three outer electrons 6s25d1. These elements belong to the 5d transition series, which explains their great similarity. On the other hand, the filling up of the 4f orbitals with one electron at a time gives them a distinctive personality. Just as Mendeleev predicted the existence of yet unknown elements based on the consequences of the periodic law, so did Jan Scholten anticipate the clinical picture of the remedies obtained from the lanthanides and their salts based on the peculiarity of their electronic structures. He had tested the validity of this approach by applying it to the already known mineral remedies [**4,5**].

 Jan Scholten performed an exercise regarding pattern recognition, a technique so modern in scientific research and practice and yet so old in reality, if we recall the theory of signatures and, to go even further back in time, Plato’s shadows.

 What homoeopathic pharmacology does, in essence, is to escape from the empire of quantity and step into the realm of quality. Quite impressive to think that this transmutation actually takes place in a kitchen-like environment with the help of humble tools, a mortar and a couple of bottles, a little water and very minute quantities of 70% ethyl alcohol.

 The above-mentioned do in no way diminish the greatness of the work of Dr. Jan Scholten, homeopath and chemist in Utrecht, Netherlands, and that of pharmacist Robert Münz in Eisenstadt, Austria. The former had the vision, the latter prepared the remedies. In some cases, the pharmacist had to resort to military laboratories in order to obtain, under strict escort, the ludicrously small quantities needed to prepare the remedy.

 Once prepared, the remedy presents a tremendous potential. For itself alone it will never be of any use. It is solely of use when it meets the living organism found in an analogous or, in other words, similar state. In addition, its use translates into healing.

 In 1993, Jan Scholten’s book Homoeopathy and Minerals appeared, exposing the connection between the clinical characteristics of mineral remedies (i.e. those obtained from proofs on healthy human subjects, from toxicological data and from healing cases) and the chemical characteristics of the substances involved in these remedies. Also, estimates were made regarding the weight of the element in its elemental form as well as regading the weight of the accompanying anions in the form of salts. Basically, Jan Scholten discovered a grammar and translated it from the language of chemistry to that of clinical medicine.

 This grammar is in fact the theory of elements, 3 which relate the 7 series (periods) of the periodic table to as many levels/phases in human life, while the periodic table groups become as many stages in the natural evolution of each level. By natural evolution, we mean the Gaussian curve that describes the emergence, the growth, the peak and the decline of a phenomenon.

 The 7 series are named after what Scholten considers to be the most representative element of the series:

 1. Hydrogen Series Theme: Being, Incarnation, Inception 

2. Carbon Series Theme: Ego and Individuation

 3. Silicium Series Theme: Family, Home, Friends, Relationships, Society 

 4. Ferrum Series Theme: Work, Task, Profession

 5. Silver Series Theme: Creativity 

 6. Gold Series Theme: Power, Leadership

 7. Uranium Series Theme: Weightlessness 

 There are 18 stages corresponding to the 18 columns of the periodic system. They are cyclic, and can be viewed as an ouroboric spiral, where decline in a series ushers a new beginning in the next series.

 Stage 1: Begin

 Stage 2: Place settle

 Stage 3: Search

 Stage 4: Establish

 Stage 5: Prepare

 Stage 6: Prove

 Stage 7: Train, cooperate

 Stage 8: Persevere

 Stage 9: Fulfill, success

 Stage 10: Supremacy, rule

 Stage 11: Keep, preserve

 Stage 12: Divide

 Stage 13: Withdraw

 Stage 14: Formal

 Stage 15: Lose

 Stage 16: Memory

 Stage 17: End, abandon

 Stage 18: Pause, rest before a new cycle

 There is always an element corresponding to the present state of the subject in its history at the crossing point between series and stage. In addition, if the subject’s problematic is to be searched in the mineral kingdom, then we have just found half of the solution: that is the similar element. The other half is the salt of the element when its elemental form is not sufficient to cover the picture of the subject’s need.

**Fig. 5:**
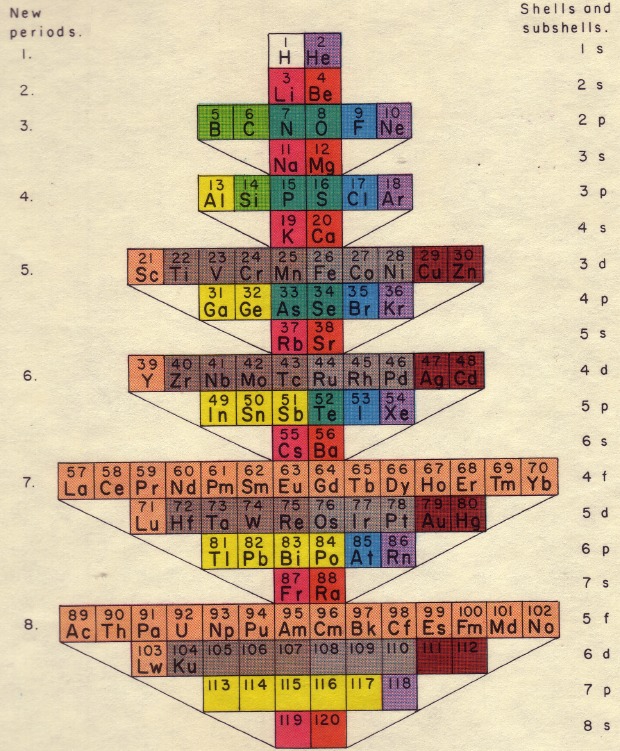
Mazur’s Periodic Table

 Lanthanum and the lanthanides would seem to correspond to the Gold series [**6**], as shown in the flat image of the periodic table. However, if we use an image that is closer to the depth of things, we will see that the lanthanides, like the actinides, present a special situation deriving from their essential characteristic of filling up the 4f and 5f orbitals, respectively. Hence, the lanthanides should be considered otherwise than part of the Gold series. The individuality of the lanthanide group is evident in all the above layouts of the periodic table, as well as in the one below (**Fig. 5**).

The characteristics of Lanthanides and their corresponding clinical behavior show that "they are different": we could say they truly bring out the third dimension; therefore their life is not lived in the "plane". By investigating their profound nature, we will notice that they do not fit into the theme of power over others and leadership. Alternatively, at least, these Gold series themes follow quite the opposite direction in the case of lanthanides. The struggle for power over others, dominance over otherness, peculiar to the Gold series, is reversed by the lanthanides into the opposite direction: mastery of the self, not over others, self- independence and self-government expressed as autonomy. Lanthanide problematic is about acquiring, preserving and controlling the self. The Self is their battlefield, their aspiration horizon. To dominate or be dominated, to own or be owned is what they abhor the most.

 The use of minerals as seen through the lens of the theory of elements, an approach initiated by Scholten in his 1993 book Homoeopathy and Minerals, continued, enriched and nuanced in Homoeopathy and the Elements (1996), reaches its acme with Secret Lanthanides in 2005.

Why acme, you may ask? Because this entire path, strengthened by accumulated experimental and clinical confirmations from colleagues who have followed his footsteps since 1994 up until now [**6-40**], made possible the sudden introduction of a whole group of elements and compounds into the therapeutic arsenal of homoeopathy: the lanthanides. This is an unprecedented fact in the history of the homoeopathic medicine.

## Conclusions

The reliability of the method of translating the physicochemical language into the clinical language while keeping the same "grammar" made possible the approximation of the clinical picture of a remedy based on its physical and chemical behavior. This approximation is sufficient to justify and guide the clinical use of the remedy, and to open the door of experience.

 It is interesting to note that remedies derived from the lanthanides address both a specific human typology and a subtle and, at the same time, a powerful pathology.

 The human typology in question is one for which the quest for the self and its meaning in the universe is truly important, overwhelming at times, with a great desire for independence and autonomy, often going through unsustainable contradictions. These contradictions are reflected in the noble tissue conditions, which often have a functional nature, such as central coordination disorders or lesion-functional, such as autoimmune processes.

 Having attended Dr. Jan Scholten’s seminars on lanthanides prior to the release of Secret Lanthanides and having acquired the lanthanide-based homeopathic remedies kit from Robert Münz Remedia Pharmacy in Eisenstadt, I have enjoyed the chance of using them ever since 2003.

 As an endocrinologist, I was daily confronted with pathologies such as autoimmune thyroiditis, a situation where the body produces antibodies against its own structures: these cases were unexpectedly solved with the help of lanthanide-compound-based remedies.

 As a homoeopath, the central nervous system coordination disorders such as dyslexia, various disorders of striated and smooth muscle, growth delays and distortions have often found support in a lanthanide-based treatment.

 The cases solved have strengthened my conviction that the lanthanides as homeopathic remedies came at the right time to meet the stressing human needs of our era and find a solution for them right here, in the 4f series of the periodic table.
